# Trust, but Verify: A “Just Culture” Model for Oversight of Potentially High-Risk Life Sciences Research

**DOI:** 10.1089/apb.2024.0053

**Published:** 2025-06-05

**Authors:** Ben C. Snyder, Joshua M. Wentzel, Gerald L. Epstein, Robert P. Kadlec, Gerald W. Parker

**Affiliations:** ^1^ Biosecurity and Pandemic Policy Center, Bush School of Government and Public Service, Texas A&M University, Washington, DC, USA.; ^2^ Meselson Center, RAND Corporation, Arlington, Virginia, USA.; ^3^ Independent Researcher, Alexandria, Virginia, USA.; ^4^ Global One Health, College of Veterinary Medicine and Biomedical Sciences, Texas A&M University, College Station, Texas, USA.

**Keywords:** just culture, non-punitive reporting, pathogens with pandemic potential, dual-use research of concern, enforcement continuum, national strategy/policy

## Abstract

**Introduction:**

A “just culture” balances accountability for misconduct with transparency, collaboration, and proactive engagement to address the root causes of accidents and compliance issues. Fostering a just culture in the oversight of potentially high-risk life sciences research would allow the U.S. government to improve biosafety and biosecurity while promoting beneficial research.

**Discussion:**

The authors explore four proposed elements of a just culture approach to oversight: (1) an enforcement continuum with options ranging from cooperative engagement to legal penalties, (2) non-punitive reporting of safety and security incidents, (3) technical assistance for researchers and institutions subject to oversight, and (4) outreach and two-way information sharing across the life sciences research enterprise.

**Conclusion:**

Promoting a just culture would support effective risk management by combining bottom-up scientific responsibility with top-down oversight and accountability. Implementing this approach will be challenging and require extensive stakeholder engagement, but adoption in the aviation industry shows it can succeed.

## Introduction

To improve biosafety and biosecurity while strengthening the life sciences research enterprise, U.S. government biorisk management policies should foster a “just culture” of oversight, especially for the small subset of life sciences research that constitutes dual-use research of concern (DURC) or involves pathogens with pandemic potential (PPP).

As defined by an international group of aviation safety experts, a just culture is “an atmosphere of trust in which people are encouraged, even rewarded, for providing essential safety-related information, but in which they are also clear about where the line must be drawn between acceptable and unacceptable behavior”.^1^ The group notes that the concept of just culture developed as a compromise between so-called “blame culture,” which holds individuals accountable for all mistakes regardless of systemic problems, and “no-blame culture,” which holds systems accountable for all mistakes regardless of individual misconduct.

A proactive, safety-first culture has long been considered a core feature of high reliability organizations (HROs)—such as U.S. Navy aircraft carriers, nuclear power plants, and air traffic control centers—that are exposed to the risk of catastrophic errors and cannot operate without maintaining a near-perfect safety record.^2^ Some efforts to implement findings from the study of HROs identify just culture as a vital component, particularly in the aviation and healthcare industries.^3^

A just culture aims to increase information exchange, uncover systemic vulnerabilities, and encourage rapid action to address compliance problems. The U.S. aviation industry, which has seen accident rates fall almost every year for the last decade, illustrates the value of this approach.^4^ Since 2015, the Federal Aviation Administration (FAA) has explicitly integrated the idea of just culture into its compliance philosophy:

“The FAA’s approach to compliance furthers the evolution toward a ‘just culture.’ […] A ‘just culture’ allows for due consideration of honest mistakes, especially in a complex environment […]. But even unintentional errors can have a serious adverse impact on safety, so we must ensure that the underlying safety concern is fixed every time”.^5^

Like operating in these environments, conducting DURC or PPP research could result in hazards to public safety or national security. A just culture approach can reconcile the demand for additional oversight with the recognition that collaboration on system-level improvements is necessary for these measures to succeed. Given the current polarization of debates around overseeing infectious disease research, this vision may offer a constructive path forward for strengthening top-down oversight while fostering a bottom-up culture of scientific responsibility and institutional accountability.

Elements of both top-down and bottom-up approaches are essential for robust, effective risk management. Without top-down governance, implementation will likely be inconsistent across institutions and malicious or irresponsible actors may escape scrutiny. Without bottom-up engagement, policies could end up being crafted without input or support from those most directly affected, resulting in burdensome and ineffective policies that could not only impede research but also hinder the development of a culture that proactively addresses the underlying drivers of risk.

The U.S. biorisk management framework, including DURC and PPP research oversight policies, should cultivate a just culture. The framework should feature an enforcement continuum with options ranging from cooperative engagement to legal penalties. Other important aspects include a non-punitive incident reporting system, technical assistance for researchers and institutions subject to oversight, and outreach and two-way information sharing across the life sciences research enterprise.

## Enforcement Continuum

Enforcement under a just culture recognizes that risks result from both individual and system-level failures and aims to collaborate with good-faith actors to address the root causes of compliance issues while preventing abuse.

Most researchers and research institutions strive to comply fully with all applicable policies and take pride in upholding the highest standards of safety and security. The limited data available about safety incidents in high-containment laboratories suggests that although inadvertent exposures and other accidents occur regularly, exceedingly few lead to community outbreaks. Even so, the worldwide growth of high-containment lab capacity in the absence of consistent biosafety and biosecurity standards will increase the risk of laboratory-acquired infections. For PPP research, the consequences of an accidental escape—or a security breach—could be catastrophic.

Although DURC may not raise the same safety concerns, the knowledge it generates poses other, more insidious risks. If someone were to publish the genetic sequence of a novel virus with pandemic potential or methods to more easily resurrect a demon from the freezer such as the variola virus, there would be no way to remove that information from the internet. The knowledge would then be permanently available to adversarial state and nonstate actors interested in biological weapons.

Biorisk management policies must be enforced in a way that rapidly fixes compliance issues, denies access to malicious and irresponsible actors, and deters their misconduct. The imperative to identify and correct problems early on, well before a major accident or publication of problematic dual-use research, necessitates collaboration with the research community.

While U.S. oversight of life sciences research relies on a fragmented patchwork of regulations and requirements tied to Federal funding, Canada has a unified set of legally enforceable regulations, including provisions related to dual-use research.^6^ Despite the potential for increased compliance costs, Canada is a leader in both oversight of the life sciences and research productivity, and the Canadian government’s use of an enforcement continuum may help explain this success. According to its biosafety and biosecurity regulator:

“Compliance is normally achieved through a cooperative approach […]. Correcting non-compliance can often be achieved through the development of appropriate corrective measures or other methods. However, when this cooperative approach does not lead to compliance, or when the regulated party is incapable of correcting non-compliance, enforcement actions may be used. In some cases, enforcement actions may be the appropriate initial tool to correct or prevent non-compliance”.^7^

Modeled on the Canadian system and the FAA’s philosophy, U.S. DURC and PPP research oversight policies should include an enforcement continuum with options ranging from cooperative engagement (e.g., support for root cause analysis of an identified issue) to legal penalties (e.g., fines). This enforcement continuum should treat most compliance problems as learning opportunities rather than evidence of wrongdoing. It should also address potential problems earlier and more quickly by leveraging the option to take actions with a lower cost than administrative or legal proceedings. Scholars have documented the success of similar, less confrontational approaches to enforcement by some European regulators.^8^

That said, out-of-compliance individuals and institutions should be held accountable for returning to compliance as soon as issues are identified. In the case of refusals, delays, repeated issues, or demonstrated inability to comply, enforcement should escalate incrementally to include administrative and legal penalties. Deliberate misconduct or reckless behavior should result in immediate escalation to punitive measures.

## Non-Punitive Reporting

As part of a just culture, a national non-punitive biosafety and biosecurity incident reporting system would serve a crucial role in promoting the exchange of information required to successfully mitigate risks. Reporting could flag compliance problems that might otherwise go unnoticed, identify gaps in existing requirements, and build the evidence base for what practices do and do not work. Several groups of experts have previously discussed or recommended non-punitive reporting systems for the life sciences.^9,10^

Biosafety and biosecurity measures have evolved primarily in response to the experiences of individual researchers and institutions rather than systematic study of the whole enterprise. Many foundational tenets date back to research performed under the former U.S. biological weapons program, which was terminated in 1969. As a result, Federal guidelines and prescriptions developed piecemeal, likely resulting in some requirements that may only provide minimal safety or security benefits compared to their cost. Furthermore, requirements must anticipate and quickly adapt to the challenges brought by rapid technological advances. Reporting minor incidents and near-misses could help identify emerging risks before they result in a consequential failure.

To cultivate trust and incentivize use, reporting systems should include protections for the individuals and institutions involved. Some universities that have already implemented non-punitive or “no-fault” reporting systems, such as the University of Chicago, offer the option to submit reports anonymously.^11^ Provided that reports can still be authenticated, a national system should preserve this option.

Nonetheless, consistent with the enforcement continuum discussed above, many compliance issues identified through reports should receive immediate, non-punitive follow-up to correct the problem. In cases of negligence or deliberate misconduct, reporting should offer no protection. A national reporting system must also consider mechanisms to prevent false or misleading accusations aimed at shutting down research or damaging the reputations of researchers or research institutions.

## Technical Assistance

A just culture aims to empower individuals to proactively identify and resolve compliance challenges. The U.S. government must ensure that the research community understands and knows how to fulfill this responsibility.

Conversations with stakeholders across the life sciences research enterprise have identified a strong interest in on-demand advice from the U.S. government about biosafety and biosecurity. Both the policy landscape and the range of possible safety and security challenges are complicated, and companies and laboratories often face uncertainty about how to respond to unfamiliar or unexpected situations.

The original charter of the National Science Advisory Board for Biosecurity (NSABB), a Federal advisory committee administered by the U.S. National Institutes of Health, charged the board with serving as an on-call source of technical assistance for institutions assessing the risk of new or especially complex dual-use experiments.^12^ Unfortunately, this vision was never fully realized; the NSABB was asked to advise on only a handful of projects, and even then it was after the research had been conducted and the results were awaiting publication. In 2012, after the NSABB attracted controversy by initially recommending against the publication of two experiments that made strains of H5N1 influenza transmissible between ferrets, this provision was dropped entirely from its charter.

More than 10 years later, some Federal agencies do offer technical assistance on biosafety and biosecurity, but there remains no centralized point of contact for compliance questions and no resources allocated for providing advice on the conduct of DURC or PPP research. Technical assistance should be provided in response to requests from entities subject to oversight, with legal protection for acting in accordance with guidance received.

The Department of Commerce’s Bureau of Industry and Security frequently responds to requests from gene synthesis providers about whether certain sequences are covered by export controls. The Federal Select Agent Program also appears to be moving toward a collaborative model of information sharing with the regulated community. Both initiatives have been welcomed by members of the communities subject to these regulations.

Future policies should expand upon these efforts with the goal of not only clarifying policy definitions but also contributing to the body of technical knowledge about the evolving risks and benefits of infectious disease research. Scientific and technological advances will continue to generate new and unforeseen edge cases outside of institutions’ existing know-how. A central clearinghouse with internal subject matter experts and trusted relationships with national security and life sciences specialists is needed to help navigate these questions until formal policymaking processes can catch up.

## Outreach and Active Engagement

In a just culture, oversight should be accompanied by increased outreach and two-way engagement across the life sciences research enterprise. This engagement is needed to build trust and cultivate widespread awareness of the importance of biorisk management, both of which are foundational for high levels of compliance and collaboration.

In addition to providing education on compliance, engagement should strengthen norms for the safe, secure, and ethical conduct of potentially high-risk research and gather information from practitioners about on-the-ground realities. This could include representation at academic and industry conferences, voluntary site visits, and educational events.

Although researchers and research institutions typically take compliance seriously, biosafety and biosecurity are rarely their primary responsibilities, and awareness about these issues varies widely. Furthermore, many members of the life sciences research enterprise may not realize they can make a positive contribution to biosecurity. Better empowering laboratory support staff to identify safety issues or signs of misuse could offer an additional measure to reduce the risk of biological incidents. Similarly, some biotechnology product and service providers, such as manufacturers of the advanced personal protective equipment needed for work at high-containment levels, might benefit from information about identifying suspicious customers and preventing misuse of their products.

Engagement efforts provide a valuable opportunity to solicit feedback from the community about the impact and effectiveness of existing rules and processes. They can also help government oversight bodies keep up to date with best practices in the field and observe the challenges and opportunities introduced by technological advances.

Finally, education and outreach should extend beyond the life sciences community to foster public transparency and public trust. This should involve publishing information about laboratory incidents, including safety measures already in place and changes made to reduce the risk of future incidents. The University of Texas Medical Branch, which has maintained a public database of all potential exposures in its research laboratories since 2002, provides an excellent example of how this approach can work in practice.^13^ Public engagement should also raise awareness about the benefits of life sciences research and the important role of high-containment laboratories in public health, medicine, and basic science.

## Conclusion

Promoting a just culture of compliance would combine favorable elements of top-down oversight and bottom-up responsibility—with accountability throughout the research enterprise—to create a more resilient biorisk management framework. A just culture prioritizes collaboration to address system-level risks while recognizing that stronger enforcement measures are sometimes needed.

It will be difficult to strike this balance while closing longstanding oversight gaps and creating a policy framework capable of adapting to future technological advances. The proposals articulated above represent a first pass at imagining such an effort, and extensive stakeholder engagement is needed to stress-test these ideas and navigate the many trade-offs involved. The global nature of these challenges makes it critical to develop governance tools that will be adopted worldwide. This is an opportunity for the U.S. to lead by example.

Some have proposed an independent authority with clear ownership of the U.S. government’s biosafety and biosecurity mission.^14,15^ The establishment of an independent authority would provide an opportunity to facilitate the shift in culture and practices required to operationalize a just culture approach. Adopting and fully implementing these principles would facilitate a cooperative transition that involves the research community, the public, and government authorities. Undoubtedly, moving toward a just culture of oversight and creating a new Federal agency are bold propositions, but they are not unprecedented. Organizations that have confronted the possibility of catastrophic failures for decades, such as the FAA, recognize a just culture and independent oversight as essential features for ensuring their safety and security.
